# Antibacterial Action of Curcumin against* Staphylococcus aureus*: A Brief Review

**DOI:** 10.1155/2016/2853045

**Published:** 2016-11-13

**Authors:** Sin-Yeang Teow, Kitson Liew, Syed A. Ali, Alan Soo-Beng Khoo, Suat-Cheng Peh

**Affiliations:** ^1^Sunway Institute for Healthcare Development (SIHD), Sunway University, 47500 Bandar Sunway, Selangor Darul Ehsan, Malaysia; ^2^Molecular Pathology Unit, Cancer Research Centre (CaRC), Institute for Medical Research (IMR), 50588 Jalan Pahang, Kuala Lumpur, Malaysia; ^3^Advanced Medical and Dental Institute (AMDI), Universiti Sains Malaysia (USM), 13200 Kepala Batas, Pulau Pinang, Malaysia; ^4^Institute for Research, Development and Innovation, International Medical University (IMU), 57000 Bukit Jalil, Kuala Lumpur, Malaysia; ^5^Anatomical Pathology Department, Sunway Medical Centre, 47500 Bandar Sunway, Selangor Darul Ehsan, Malaysia

## Abstract

Curcumin, the major constituent of* Curcuma longa* L. (Zingiberaceae family) or turmeric, commonly used for cooking in Asian cuisine, is known to possess a broad range of pharmacological properties at relatively nontoxic doses. Curcumin is found to be effective against* Staphylococcus aureus* (*S. aureus*). As demonstrated by* in vitro* experiment, curcumin exerts even more potent effects when used in combination with various other antibacterial agents. Hence, curcumin which is a natural product derived from plant is believed to have profound medicinal benefits and could be potentially developed into a naturally derived antibiotic in the future. However, there are several noteworthy challenges in the development of curcumin as a medicine.* S. aureus* infections, particularly those caused by the multidrug-resistant strains, have emerged as a global health issue and urgent action is needed. This review focuses on the antibacterial activities of curcumin against both methicillin-sensitive* S. aureus* (MSSA) and methicillin-resistant* S. aureus* (MRSA). We also attempt to highlight the potential challenges in the effort of developing curcumin into a therapeutic antibacterial agent.

## 1. Introduction

Curcumin or diferuloylmethane is the major phytochemical of* Curcuma longa* L. (Zingiberaceae family), which is commonly known as turmeric. Curcumin is the polyphenolic compound that gives the yellow colour of the herb. Turmeric is mainly cultivated in tropical and subtropical regions and is mainly produced by India. Traditionally, it has been used to flavour food, dye cloths, and treat various human ailments [1]. Curcumin is extracted from turmeric by solvent extraction (preferably with ethanol) through various methods (e.g., Soxhlet, ultrasonic, microwave, and supercritical carbon dioxide) followed by purification via column chromatography [2, 3]. Ever since the identification of curcumin as the main constituent of turmeric, multiple pharmacological activities of curcumin that include antimicrobial, antidiabetic, anti-inflammatory, anticancer, and antioxidant have been reported [4–6]. More excitingly, when combined with other drugs, curcumin has been found to enhance the effects of antibacterial [7–9], antifungal [10, 11], anticancer [12, 13], and antioxidant [14, 15] activities.

Curcumin usually exhibits low to no toxicity at the active doses. A systematic review from the MEDLINE computerized database (1966 to 2002) has shown that curcumin is safe when consumed up to 8 g each day consecutively for 3 months in a phase I human trial that involved 25 subjects [16]. Similarly, the dose of 8 g per day was safe when used in combination with gemcitabine that showed marked therapeutic effects in pancreatic cancer patients [17, 18]. Interestingly, curcumin is also able to reverse the Aflatoxin B1-induced toxicity and iron-overloaded liver toxicity in rats [19–21]. Despite being extensively studied, the exact mechanism(s) of curcumin's multiple biological and pharmacological activities remains to be explored. Based on the available literature, there are two hypotheses describing the poly-pharmacological effects of curcumin. First, curcumin is known to act on multiple targets [4, 5, 22–24], hence having diverse roles in regulating various cellular processes. Secondly, products resulting from the curcumin degradation have been shown to be highly diverse depending on the chemical or biochemical reactions involved [25–27]. Most of these products are stable and function differently that may lead to the multiple effects.

The most studied activity of the curcumin in the past 10 years is the anticancer effects [28]. However, the first paper describing the biological action of curcumin was its antibacterial activity against various bacteria:* S. aureus*,* Trichophyton gypseum*,* Salmonella paratyphi*, and* Mycobacterium tuberculosis* [29]. To date, studies on the antibacterial activity of curcumin that indicate inhibition properties of a wide range of bacteria are increasingly documented [6, 23, 30]. Recent publications have also reported that curcumin is active against a plethora of drug-resistant bacterial strains [8, 9, 31, 32].* S. aureus* infection is a major problem in many developing countries, especially in hospitals where the MRSA spreading is difficult to control [33]. Over the years, the multidrug-resistant* S. aureus* infection has increased the global morbidity and mortality [34, 35]. Due to the difficulty in treating the infection, it has consequently imposed an elevating burden on healthcare resources [36–38]. Cumulative findings in recent years have shown that curcumin is active against both MSSA and MRSA [8, 9, 30, 32, 39, 40]. In view of the need for a more efficacious and safe therapeutic modality towards the drug-resistant* S. aureus*, we discuss the reported antibacterial activities of curcumin against* S. aureus* and its potentials and limitations to be developed into a potent antibiotic.

## 2. Curcumin-Mediated Inhibition of* S. aureus*

Curcumin inhibits the growth of both Gram-positive and Gram-negative bacteria [6, 23, 30].* S. aureus* is one of the Gram-positive strains that is susceptible to curcumin-mediated inhibition.* S. aureus* is a pathogen that causes various infections including infective endocarditis (IE), bacteremia, skin and soft tissue, osteoarticular, and pleuropulmonary infections [33]. Over the years,* S. aureus* has evolved and developed multiple strategies to evade human immune system and to resist antibiotics treatment. This has given rise to the evolution of MRSA, and the emergence of healthcare-associated (HA) and community-associated (CA) MRSA has caused a major problem to the human society [45, 46]. In this section, we discuss the past and current works that show the curcumin-mediated killings of MSSA and MRSA (summarized in Table 1).

Mun et al. [9] showed that the minimal inhibitory concentrations (MICs) of curcumin against 10 strains of* S. aureus* (including 2 ATCC MSSA and MRSA standard strains, 4 MRSA clinical isolates, and 4 MRSA from culture collection) ranged from 125 to 250 *μ*g/mL while a study by Wang et al. [40] showed the MIC of 256 *μ*g/mL against MSSA. Using a broth microdilution assay, our group [8] also showed that 250 *μ*g/mL curcumin was required to kill the two ATCC MSSA (#25923) and MRSA (#43300) strains. However, another study demonstrated that the MICs against the ATCC standard MSSA and MRSA were 219 and 217 *μ*g/mL, respectively, that are slightly lower than the former study [47]. Recently, Kali et al. [48] showed the mean curcumin MIC of 126.9 *μ*g/mL against 15 Gram-positive bacterial isolates including thirteen* S. aureus* and two* Enterococcus faecalis*. Nonetheless, this study is not used for comparison in Table 1 because the obtained MIC might not be representative for curcumin's effect against* S. aureus* as the study was carried out in combination with* Enterococcus faecalis.*

**Figure 1:**
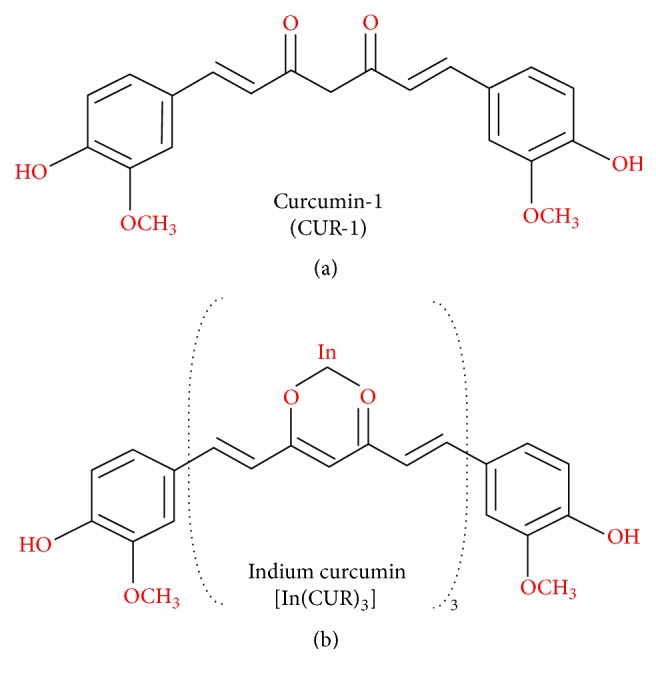
Chemical structures of antibacterial curcumin analogues against* S. aureus*. Upper panel shows the structure of curcumin-1 [30, 41] while the lower panel shows the metal complexes of curcumin, indium curcumin [42, 43]. The chemical structures above were drawn using a free online tool, ChemWriter (http://chemwriter.com/).

A more potent inhibition was achieved when curcumin-1 (CUR-1), a major component of commercial preparations of curcumin (purity > 98%), was used against* S. aureus*. The chemical structure of curcumin-1 is shown in Figure 1. Tyagi et al. [30] showed that the curcumin-1 was active against MSSA at concentration of as low as 25 *μ*M (equivalent to 9.21 *μ*g/mL), as it killed 50% of the bacteria after 2 hr incubation. The activity was time- and dose-dependent, and 100% killing was achieved at 50 *μ*M (equivalent to 18.42 *μ*g/mL) after 2 hr exposure [30]. In contrast, Sasidharan et al. [41] showed that the same compound had a MIC of 250 *μ*g/mL against* S. aureus*, which is comparable to the native curcumin [8, 9]. In an* in vivo* mouse model, administration of 100 mg/kg curcumin was shown to protect the mice infected with both MSSA and MRSA from pneumonia by targeting the *α*-hemolysin (HIa) protein of* S. aureus* [40]. In summary, the curcumin MICs against* S. aureus* ranged from 18.42 to 256 *μ*g/mL (refer to Table 1). The variation could be due to (i) strain difference (i.e., MRSA versus MSSA); (ii) source of bacterial strains (i.e., ATCC standard strains versus clinical isolates); (iii) type of antibacterial assay (i.e., disk diffusion versus broth microdilution); and (iv) type of curcumin and its solvent (i.e., commercial compound versus in-house purified compound). Overall, the cumulative findings showed that there is no difference of MICs against MSSA and MRSA, suggesting that the sensitivity towards curcumin treatment is not altered by the multidrug resistance machinery in* S. aureus*.

**Figure 2:**
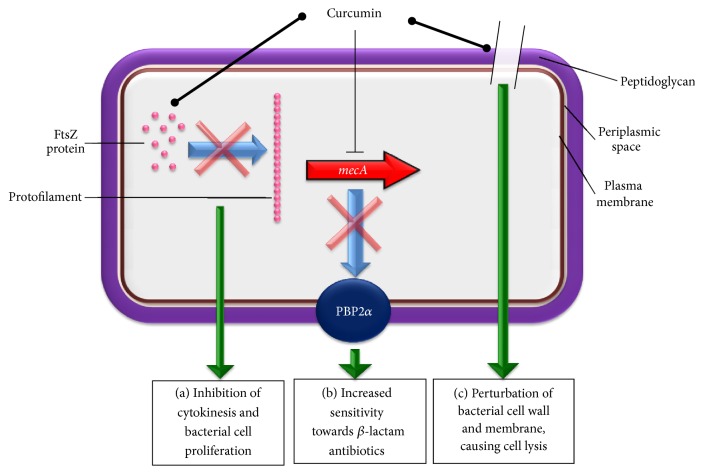
The potential mechanisms underlying the antibacterial effect of curcumin against* S. aureus*. Circle-shaped arrow indicates binding whereas blocked arrow represents inhibition. (a) Curcumin may bind into FtsZ proteins, thereby inhibiting the assembly of FtsZ protofilaments. This, in turn, suppresses the formation of Z-ring leading to inhibition of cytokinesis and bacterial proliferation [44]. (b) In the case of MRSA, curcumin could inhibit the* mecA* gene transcription, causing reduced expression of PBP2*α* proteins. As a result, MRSA can be sensitized towards the antibacterial action of *β*-lactam antibiotics such as Penicillin and Methicillin [32]. (c) The binding between curcumin and peptidoglycan on* S. aureus* cell wall could trigger damage on the cell wall and membrane, leading to cell lysis of* S. aureus* [30, 32].

There have been several explanations on how curcumin acts and kills the bacteria which are illustrated in Figure 2. Rai et al. [44] have demonstrated that curcumin interacts with FtsZ (prokaryotic homologue of eukaryotic cytoskeletal protein tubulin)* in vitro* and inhibits the assembly of FtsZ protofilaments in* Bacillus subtilis* 168. Although it has not been examined directly on* S. aureus*, it is believed that inhibiting the assembly dynamics of FtsZ is one of the main mechanisms of curcumin in inhibiting bacterial cell proliferation. FtsZ is also believed to be a novel target for the development of antibacterial drugs against* S. aureus*. [63, 64]. Mun et al. [32] showed that the antibacterial action of curcumin against both MSSA and MRSA was markedly enhanced when used in combination with ATPase inhibitors and mild detergents that compromise ATP-binding cassette (ABC) transporters and cytoplasmic membrane integrity, respectively. The same study has also shown that curcumin binds to peptidoglycan (PGN), and the increasing concentrations of PGN block the curcumin antibacterial activity. Tyagi et al. [30] also showed that curcumin-1 inhibited* S. aureus* growth by perturbing the bacterial membrane integrity. In this study, the bacterial membrane of* S. aureus* was examined using two fluorescent probes: propidium iodide and calcein. The membrane leakage upon exposure to curcumin was also evaluated by fluorescence and scanning electron microscopies. Although existing evidence suggests that curcumin inhibits* S. aureus* mainly by damaging the bacterial membrane, further investigation is required to identify additional bacterial target proteins besides FtsZ and PGN. This is important not only to enhance the understanding of the curcumin interaction with its target proteins, but also to further improve the activity of curcumin against* S. aureus*, particularly the MRSA strains. Similar studies should also be performed on the curcumin derivatives such as curcumin-1 (curcumin with highest purity >98%) and indium curcumin (metal complex with curcumin) which have shown more potent antibacterial effects than the native curcumin. The chemical structures of curcumin-1 and indium curcumin are shown in Figure 1.

## 3. Synergism of Curcumin with Antibiotics against* S. aureus*

In addition to showing potent antibacterial activity when used alone, curcumin also exerts marked activity against* S. aureus* when used at subinhibitory dose in combination with various other antibiotics [8, 9, 31, 32]. These findings are interesting since curcumin is naturally derived from turmeric, which is one of the major ingredients of Asian cuisine [1, 23]. Of note, crude turmeric extracts have previously shown marked antibacterial activities against* S. aureus* [6, 65, 66]. This section discusses the synergistic antibacterial activity of curcumin with antibiotics against MSSA and MRSA.  Table 2 summarizes studies that demonstrated the synergism of antibiotics-curcumin. In this table, we include the information of curcumin type, solvent, and concentration, type of bacteria-killing assay, and* S. aureus* strains for comparison.

The first study that reported the synergism of curcumin against* S. aureus* was seven years ago by Moghaddam et al. [31]. Using a disk diffusion method, Moghaddam et al. [31] showed that 500 *μ*g/disc of curcumin exerted synergistic antibacterial effect on the MSSA when used in combination with four antibiotics. Combination with cefotaxime, cefixime, tetracycline, and vancomycin resulted in increase of inhibition zone of 52.6, 24.9, 26.5, and 24.4%, respectively. This was then followed by Mun et al. [9] study in 2013 that showed curcumin's synergism with Oxacillin, Ampicillin, Ciprofloxacin, and Norfloxacin against the MRSA. This study employed checkerboard broth microdilution assay method to determine the synergistic activity. This method allows more standardised testing and is less laborious and suitable for studies with multiple concentrations of test compounds [67]. Other studies also showed the synergistic antibacterial effect of curcumin against both MSSA and MRSA when used in combination with antibiotics such as Cefaclor, Cefodizime, Cefotaxime, Gentamicin, Amikacin, Penicillin, and Erythromycin (refer to Table 2). Based on reported findings, the curcumin synergism in combination with antibiotics appears to be relatively nonspecific. Antibiotics classes that have shown synergism with curcumin include *β*-lactams, Cephalosporins, Aminoglycosides, Glycopeptides, Tetracyclines, and Fluoroquinolones. This might due to the multitargeting action of curcumin or their undetermined breakdown products as pointed out in the previous section. More studies are needed to evaluate the mechanism of curcumin synergism based on the different classes of antibiotics. In addition to antibacterial action, curcumin also reverses the drug resistance when used in combination with other anticancer agents such as cisplatin, 5-fluorouracil, oxaliplatin, and doxorubicin in multiple types of cancer cells including breast [68], colon [69], head and neck [70], and ovary [71]. The curcumin may have acted on the target or pathway related to the development of drug resistance, hence restoring the killing effect of the drugs [72, 73]. This may be one of the mechanisms on how curcumin enhances the effect of antibacterial drugs, especially when they are targeting* S. aureus*-infected human cells.

In recent years, MRSA infection has emerged as a serious infection due to its multidrug resistance (MDR) especially in the hospital setting [74]. The MRSA infection may spread rapidly especially when the disease is not well controlled. Curcumin exhibits potent activity against MRSA, not only when used alone, but also in combination with other antibiotics. In an effort to understand the curcumin's anti-MRSA effect, Mun et al. [32] showed that the Tris and Triton X-100 inhibited the bacterial growth to 63% and 59%, respectively, when used together with curcumin. This suggests that bacterial membrane permeability is partly responsible in regulating the antibacterial efficacy of curcumin against MRSA. The same group has also shown that ATPase inhibitors (DCCD and NaN_3_) which block the ATP-binding cassette (ABC) enhanced the MRSA killing when used together with curcumin. The importance of membrane permeability/integrity in curcumin effect was also confirmed when the increase of peptidoglycan (PGN) concentration successively blocked the curcumin antibacterial activity [32]. These findings suggest that any drug or inhibitor that primarily acts on the bacterial membrane has higher chance of showing enhanced activity when used together with curcumin. It is also noteworthy that the expression of Penicillin-binding protein 2*α* (PBP2*α*), a protein responsible in conferring resistance towards *β*-lactam antibiotics, was downregulated in MRSA upon curcumin treatment [32]. This protein which is encoded by* MecA*, a nonnative gene in MRSA has significantly reduced affinity for *β*-lactam antibiotics such as Methicillin and Penicillin. Cell-wall biosynthesis, the target of *β*-lactam, could therefore carry on in MRSA despite the presence of potent doses of these antibiotics [75]. The detailed mode of action of curcumin in inhibiting PBP2*α* expression in MRSA is not clear at this juncture. It is plausible that curcumin may act on the transcription of* MecA* gene, thereby blocking the expression of PBP2*α* protein (Figure 2).

In addition to antibiotics, there are evidences showing that curcumin augments the activity of other natural compounds against MSSA and MRSA. Balan et al. [76] demonstrated that combination of curcumin and whey proteins markedly inhibited* S. aureus* growth* in vitro*. Sharma et al. [77] have also previously reported the combination effect of curcumin with several phytochemicals such as cinnamaldehyde, ellagic acid, and eugenol against* Staphylococcus epidermidis* (*S. epidermidis*), which is closely related to* S. aureus*. The combination activities as such against* S. aureus* remain to be investigated. Indeed, there have been many studies showing the potent antibacterial action of other natural compounds against* S. aureus* such as thymoquinone [78], rhein [79], emodin [80], silibinin [81], osthol [82], tannic acid [83], and epigallocatechin gallate [84]. These findings warrant the potential use of abovementioned compounds in combination with curcumin against* S. aureus*.

## 4. The Challenges of Using Curcumin as Antibiotic

Cumulative findings suggest that curcumin has broad-spectrum antibacterial activities and has synergistic effects with other antibiotics in combination therapies* in vitro* [1, 6, 85]. Curcumin has also shown potent antibacterial action in the mouse model [40]. Nonetheless, the curcumin antibacterial activity has never been evaluated in clinical trials with an aim of using it as a future antibiotic. In this section, we discuss the underlying challenges from the clinical perspectives in developing curcumin into a potential antibiotic.

While curcumin is known to possess the pharmacological activities at relatively low doses, several studies have evidenced some cytotoxicity of curcumin [50–54, 86]. The first study that demonstrated the curcumin toxicity was by Goodpasture and Arrighi [50]. They showed that turmeric resulted in an induction of chromosome aberrations in tested cell lines starting from 10 *μ*g/mL. Other studies have also shown the toxic effects of curcumin mainly on the DNA damage and chromosome aberrations [51–53]. While DNA alteration is the starting point of carcinogenesis, the use of curcumin under abovementioned conditions might be an issue. In other words, curcumin treatment may cause cancers even though the anticancer action of curcumin is well documented. In 1993, a study has concluded that turmeric oleoresin (turmeric extract containing 79–85% of curcumin) has carcinogenic property in rats and mice [54]. Mice taking 0.2 mg/kg body weight of curcumin daily average were found to have carcinomas in their small intestines. Curcumin has also shown to promote lung cancer in another study [55]. The tumour-promoting activity of curcumin has been linked to the induction of reactive oxygen species (ROS) production such as superoxide anion and hydrogen peroxide [87–89].

As curcumin is an active iron chelator, it may potentially affect systemic iron metabolism especially those who have suboptimal iron status [56]. Furthermore, curcumin has been reported to block the enzymes that metabolize drugs such as cytochrome P450s [57, 58]. This may lead to the accumulation of nonmetabolized drugs in blood and result in undesired toxicity. In human, nonetheless, the side effects of curcumin have been relatively mild. A human trial has shown that curcumin ranging from 0.9 to 3.6 g per day up to 4 months only caused some adverse effects that included nausea, diarrhea, and increased serum alkaline phosphatase and lactate dehydrogenase [90]. In 2010, Balaji and Chempakam [91] have predicted a few toxigenic and potent compounds from turmeric using a cost-effective cheminformatics approach. This method can be adopted to select the effective but nontoxic curcumin or its derivatives for further biological studies. However, the selected compound has to be evaluated in a long-term study at its active dose against* S. aureus* in order to confirm the safety of using curcumin as a potential antibiotic.

Curcumin is usually extracted from turmeric plant mainly by solvent extraction followed by column-based purification [2, 3]. Curcumin is sparingly soluble in water (<0.1 mg/mL) and is mainly dissolved in organic solvents such as DMSO, DMF, or ethanol (Tables 1 and 2). This may be the major concern when it is administered into human system as human plasma is composed of 92% of water. The water-insoluble nature may affect curcumin bioavailability and hence affect its pharmacological potential [26, 27, 61]. To this end, several methods have been developed in recent years to circumvent the poor solubility and stability of curcumin, thereby maximizing its pharmacological or biological actions. For example, it has been reported that the use of heat could enhance the curcumin solubility [59, 60]. Kurien et al. [59] have reported the improved water solubility of curcumin from 0.6 to 7.4 *μ*g/mL, without displaying heat-mediated destruction of the chemical structure. Development of curcumin bioconjugates has also shown to be an effective method of enhancing the curcumin solubility. For instances, conjugation of curcumin with hyaluronic acid formed micelles in aqueous phase at physiological pH and appeared to be nontoxic [92]. Dey and Sreenivasan have also conjugated curcumin with alginate, a natural polysaccharide product, in order to increase its stability and bioavailability [93]. Other macromolecules that could serve as carrier systems for curcumin include beta-casein [94], chitosan/Tween 20 [95], emulsomes [96], sodium caseinate [97], and albumin [98, 99]. The development of curcumin nanoformulations has been extensively reviewed in light of its anticancer action [100–102]. While increasing number of curcumin nanoformulation is being introduced into the therapeutic field, it is important to ensure that the bioconjugates or nanoformulations do not diminish the antibacterial effects of curcumin at the expense of improved bioavailability in order to develop them into effective antibiotics in the future.

In addition to potential toxicity, poor solubility, and low bioavailability, curcumin encounters multiple challenges when it is administered either through oral or intravenous route due to the nature of body system [26, 59, 61, 62]. A large amount of curcumin may get degraded in the presence of detoxifying and metabolic enzymes, or it may bind to the circulatory proteins such as albumin which may potentially reduce its activity. Contradictorily, there have been evidences showing that degraded products from curcumin are responsible of its pharmacological activities [26, 27]. Furthermore, it has been shown that albumin-bound curcumin exerted similar level of activity compared to the DMSO-dissolved curcumin in serum [103]. Of note, the curcumin degradation and binding with physiological proteins have not been evaluated in light of the curcumin antibacterial action. Whether or not these factors would affect the activity of curcumin, further investigations are required. Notably, development of the curcumin bioconjugates, nanoformulations, or derivatives could be the key to overcome the challenges mentioned above (summarized in Table 3). The development of modified curcumin has been recently reviewed [62, 100, 101].

## 5. Conclusion

Curcumin has shown potent antibacterial activity and other pharmacological actions in the past 50 years. Curcumin has been marketed globally as a health supplement mainly for its antioxidant and anti-inflammatory properties. In addition, it also has high potential to be developed into an antibiotic against* S. aureus* and other bacterial strains in the future. However, the challenges mentioned in the preceding sections should be taken into consideration to open the door for the development of more biologically active curcumin derivatives. To the best of our understanding, this is the first review that compares and summarizes the curcumin antibacterial activity against* S. aureus*. More investigation is required in order to better understand the broad action of curcumin prior to develop this compound or its derivatives into a potential antibiotic.

## Figures and Tables

**Table 1:** Antibacterial activity of curcumin against *S. aureus.*

Compound (solvent)	MIC (*µ*g/mL)	*S. aureus* strain	Test method	Reference
Curcumin (DMSO)	187.5	MSSA (ATCC 25923)	Broth macrodilution	[42]
Indium curcumin (DMSO)	93.8			

Curcumin (DMSO)	125–250	MSSA (ATCC 25923) MRSA (ATCC 33591) MRSA (4 Clinical isolates) MRSA (4 from CCARM)^#^	Broth microdilution	[9]

Curcumin-1 (DMSO)	250	MSSA (MTCC 902)^*∗*^	Broth microdilution	[41]

Curcumin (DMF)	250	MSSA (ATCC 25923) MRSA (ATCC 43300) MSSA (1 Clinical isolates) MSSA (10 Env. isolates)	Broth microdilution	[8]

Curcumin-1 (DMSO)	18.42	MSSA (ATCC 29213)	Colony counting method	[30]

Curcumin (ethanol)	219	MSSA (ATCC 29213)	Broth macrodilution	[47]
	217	MRSA (ATCC 43300)		

Curcumin (ethanol)	125	MSSA (ATCC 25923)	Broth macrodilution	[49]

Curcumin (DMSO)	256	MSSA (USA 300) MSSA (8325-4)	Broth microdilution	[40]

^#^CCARM: culture collection of antimicrobial resistant microbes.

^*∗*^Purchased from Microbial Type Culture Collection Centre (MTCC), IMTECH, Chandigarh, India.

Env.: environmental.

**Table 2:** Synergism of curcumin against *S. aureus.*

Compound (solvent)	Subinhibitory concentration	Antibiotics	*S. aureus* strain	Test method	Reference
Curcumin (not reported)	500 *µ*g/disc	Cefixime Cephotaxime Vancomycin Tetracycline	MSSA (1 clinical isolate)	Disk diffusion	[31]

Curcumin (DMSO)	Checkerboard (various serial dilutions)	Oxacillin Ampicillin Ciprofloxacin Norfloxacin	MSSA (ATCC 25923) MRSA (ATCC 33591) MRSA (1 clinical isolate)	Broth microdilution	[9]

Curcumin-1 (DMSO)	Checkerboard (various serial dilutions)	Cefaclor Cefodizime Cefotaxime	MSSA (MTCC 902)^*∗*^	Broth microdilution	[41]

Curcumin (DMF)	25 *µ*g/mL	Gentamicin Amikacin Ciprofloxacin	MSSA (ATCC 25923) MRSA (ATCC 43300) MSSA (1 clinical isolate) MSSA (10 environ. isolates)	Disk diffusion & broth microdilution	[8]

Curcumin (DMSO)	32 *µ*g/mL	Penicillin Erythromycin Ciprofloxacin	MSSA (ATCC 25923) MSSA (13 clinical isolates)	Disk diffusion	[48]

^*∗*^Purchased from Microbial Type Culture Collection Centre (MTCC), IMTECH, Chandigarh, India.

**Table 3:** Challenges of curcumin use in clinical setting.

Challenge	References
*Cytotoxicity*	
DNA damage and chromosome aberrations	[50–53]
*Carcinogenesis *	
Promote tumour formation *in vivo*	[54, 55]
*Iron chelation*	
Alter systemic iron metabolism	[56]
*Enzyme inhibition*	
Inhibit drug-metabolizing enzymes	[57, 58]
*Solubility*	
Hydrophobic nature does not support water solubility	[59, 60]
*Bioavailability*	
Degradation by plasma protease and nonspecific protein binding	[26, 27, 61, 62]

## References

[1] Goel A., Kunnumakkara A. B., Aggarwal B. B. (2008). Curcumin as ‘Curecumin’: from kitchen to clinic. *Biochemical Pharmacology*.

[2] Li M., Ngadi M. O., Ma Y. (2014). Optimisation of pulsed ultrasonic and microwave-assisted extraction for curcuminoids by response surface methodology and kinetic study. *Food Chemistry*.

[3] Priyadarsini K. I. (2014). The chemistry of curcumin: from extraction to therapeutic agent. *Molecules*.

[4] Gupta S. C., Prasad S., Kim J. H. (2011). Multitargeting by curcumin as revealed by molecular interaction studies. *Natural Product Reports*.

[5] Maheshwari R. K., Singh A. K., Gaddipati J., Srimal R. C. (2006). Multiple biological activities of curcumin: a short review. *Life Sciences*.

[6] Moghadamtousi S. Z., Kadir H. A., Hassandarvish P., Tajik H., Abubakar S., Zandi K. (2014). A review on antibacterial, antiviral, and antifungal activity of curcumin. *BioMed Research International*.

[7] Bansal S., Chhibber S. (2010). Curcumin alone and in combination with augmentin protects against pulmonary inflammation and acute lung injury generated during *Klebsiella pneumoniae* B5055-induced lung infection in BALB/c mice. *Journal of Medical Microbiology*.

[8] Teow S.-Y., Ali S. A. (2015). Synergistic antibacterial activity of curcumin with antibiotics against *Staphylococcus aureus*. *Pakistan Journal of Pharmaceutical Sciences*.

[9] Mun S.-H., Joung D.-K., Kim Y.-S. (2013). Synergistic antibacterial effect of curcumin against methicillin-resistant *Staphylococcus aureus*. *Phytomedicine*.

[10] Garcia-Gomes A. S., Curvelo J. A. R., Soares R. M. A., Ferreira-Pereira A. (2012). Curcumin acts synergistically with fluconazole to sensitize a clinical isolate of *Candida albicans* showing a MDR phenotype. *Medical Mycology*.

[11] Sharma M., Manoharlal R., Negi A. S., Prasad R. (2010). Synergistic anticandidal activity of pure polyphenol curcumin i in combination with azoles and polyenes generates reactive oxygen species leading to apoptosis. *FEMS Yeast Research*.

[12] Patel B. B., Majumdar A. P. N. (2009). Synergistic role of curcumin with current therapeutics in colorectal cancer: minireview. *Nutrition and Cancer*.

[13] Boztas A. O., Karakuzu O., Galante G. (2013). Synergistic interaction of paclitaxel and curcumin with cyclodextrin polymer complexation in human cancer cells. *Molecular Pharmaceutics*.

[14] Aftab N., Vieira A. (2010). Antioxidant activities of curcumin and combinations of this curcuminoid with other phytochemicals. *Phytotherapy Research*.

[15] Naksuriya O., Okonogi S. (2015). Comparison and combination effects on antioxidant power of curcumin with gallic acid, ascorbic acid, and xanthone. *Drug Discoveries and Therapeutics*.

[16] Chainani-Wu N. (2003). Safety and anti-inflammatory activity of curcumin: a component of tumeric (*Curcuma longa*). *Journal of Alternative and Complementary Medicine*.

[17] Kanai M., Yoshimura K., Asada M. (2011). A phase I/II study of gemcitabine-based chemotherapy plus curcumin for patients with gemcitabine-resistant pancreatic cancer. *Cancer Chemotherapy and Pharmacology*.

[18] Dhillon N., Aggarwal B. B., Newman R. A. (2008). Phase II trial of curcumin in patients with advanced pancreatic cancer. *Clinical Cancer Research*.

[19] Poapolathep S., Imsilp K., Machii K., Kumagai S., Poapolathep A. (2015). The effects of curcumin on aflatoxin B1- induced toxicity in rats. *Biocontrol Science*.

[20] Messner D. J., Sivam G., Kowdley K. V. (2009). Curcumin reduces the toxic effects of iron loading in rat liver epithelial cells. *Liver International*.

[21] Badria F. A., Ibrahim A. S., Badria A. F., Elmarakby A. A. (2015). Curcumin attenuates iron accumulation and oxidative stress in the liver and spleen of chronic iron-overloaded rats. *PLoS ONE*.

[22] Teiten M.-H., Eifes S., Dicato M., Diederich M. (2010). Curcumin-the paradigm of a multi-target natural compound with applications in cancer prevention and treatment. *Toxins*.

[23] Gupta S. C., Patchva S., Koh W., Aggarwal B. B. (2012). Discovery of curcumin, a component of golden spice, and its miraculous biological activities. *Clinical and Experimental Pharmacology and Physiology*.

[24] Zhou H., Beevers C. S., Huang S. (2011). The targets of curcumin. *Current Drug Targets*.

[25] Schneider C., Gordon O. N., Edwards R. L., Luis P. B. (2015). Degradation of curcumin: from mechanism to biological implications. *Journal of Agricultural and Food Chemistry*.

[26] Shen L., Ji H.-F. (2012). The pharmacology of curcumin: Is it the degradation products?. *Trends in Molecular Medicine*.

[27] Shen L., Liu C.-C., An C.-Y., Ji H.-F. (2016). How does curcumin work with poor bioavailability? Clues from experimental and theoretical studies. *Scientific Reports*.

[28] Ghosh D., Bagchi D., Konishi T. (2014). *Clinical Aspects of Functional Foods and Nutraceuticals*.

[29] Schraufstätter E., Bernt H. (1949). Antibacterial action of curcumin and related compounds. *Nature*.

[30] Tyagi P., Singh M., Kumari H., Kumari A., Mukhopadhyay K. (2015). Bactericidal activity of curcumin I is associated with damaging of bacterial membrane. *PLoS ONE*.

[31] Moghaddam K., Iranshahi M., Yazdi M., Shahverdi A. (2009). The combination effect of curcumin with different antibiotics against Staphylococcus aureus. *International Journal of Green Pharmacy*.

[32] Mun S.-H., Kim S.-B., Kong R. (2014). Curcumin reverse methicillin resistance in *Staphylococcus aureus*. *Molecules*.

[33] Tong S. Y. C., Davis J. S., Eichenberger E., Holland T. L., Fowler V. G. (2015). *Staphylococcus aureus* infections: epidemiology, pathophysiology, clinical manifestations, and management. *Clinical Microbiology Reviews*.

[34] Boucher H. W., Corey G. R. (2008). Epidemiology of methicillin-resistant *Staphylococcus aureus*. *Clinical Infectious Diseases*.

[35] Klein E., Smith D. L., Laxminarayan R. (2007). Hospitalizations and deaths caused by methicillin-resistant *Staphylococcus aureus*, United States, 1999-2005. *Emerging Infectious Diseases*.

[36] Tarai B., Das P., Kumar D. (2013). Recurrent challenges for clinicians: emergence of methicillin-resistant *Staphylococcus aureus*, vancomycin resistance, and current treatment options. *Journal of Laboratory Physicians*.

[37] Ventola C. L. (2015). The antibiotic resistance crisis—part 1: causes and threats. *Pharmacy and Therapeutics*.

[38] Rasmussen R. V., Fowler V. G., Skov R., Bruun N. E. (2011). Future challenges and treatment of *Staphylococcus aureus* bacteremia with emphasis on MRSA. *Future Microbiology*.

[39] Ribeiro A. P. D., Pavarina A. C., Dovigo L. N. (2013). Phototoxic effect of curcumin on methicillin-resistant *Staphylococcus aureus* and L929 fibroblasts. *Lasers in Medical Science*.

[40] Wang J., Zhou X., Li W., Deng X., Deng Y., Niu X. (2016). Curcumin protects mice from *Staphylococcus aureus* pneumonia by interfering with the self-assembly process of *α*-hemolysin. *Scientific Reports*.

[41] Sasidharan N. K., Sreekala S. R., Jacob J., Nambisan B. (2014). *In vitro* synergistic effect of curcumin in combination with third generation cephalosporins against bacteria associated with infectious diarrhea. *BioMed Research International*.

[42] Tajbakhsh S., Mohammadi K., Deilami I. (2008). Antibacterial activity of indium curcumin and indium diacetylcurcumin. *African Journal of Biotechnology*.

[43] Mohammadi K., Thompson K. H., Patrick B. O. (2005). Synthesis and characterization of dual function vanadyl, gallium and indium curcumin complexes for medicinal applications. *Journal of Inorganic Biochemistry*.

[44] Rai D., Singh J. K., Roy N., Panda D. (2008). Curcumin inhibits FtsZ assembly: an attractive mechanism for its antibacterial activity. *Biochemical Journal*.

[45] Liu G. Y. (2009). Molecular pathogenesis of *Staphylococcus aureus* infection. *Pediatric Research*.

[46] Miller L. G., Kaplan S. L. (2009). *Staphylococcus aureus*: a community pathogen. *Infectious Disease Clinics of North America*.

[47] Gunes H., Gulen D., Mutlu R., Gumus A., Tas T., Topkaya A. E. (2016). Antibacterial effects of curcumin: an *in vitro* minimum inhibitory concentration study. *Toxicology and Industrial Health*.

[48] Kali A., Bhuvaneshwar D., Charles P. V., Seetha K. (2016). Antibacterial synergy of curcumin with antibiotics against biofilm producing clinical bacterial isolates. *Journal of Basic and Clinical Pharmacy*.

[49] Sandikci Altunatmaz S., Yilmaz Aksu F., Issa G., Basaran Kahraman B., Dulger Altiner D., Buyukunal S. (2016). Antimicrobial effects of curcumin against *L. monocytogenes, S. aureus, S. Typhimurium* and *E. coli* O157 : H7 pathogens in minced meat. *Veterinární Medicína*.

[50] Goodpasture C. E., Arrighi F. E. (1976). Effects of food seasonings on the cell cycle and chromosome morphology of mammalian cells in vitro with special reference to turmeric. *Food and Cosmetics Toxicology*.

[51] Cao J., Jia L., Zhou H.-M., Liu Y., Zhong L.-F. (2006). Mitochondrial and nuclear DNA damage induced by curcumin in human hepatoma G2 cells. *Toxicological Sciences*.

[52] Urbina-Cano P., Bobadilla-Morales L., Ramírez-Herrera M. A. (2006). DNA damage in mouse lymphocytes exposed to curcumin and copper. *Journal of Applied Genetics*.

[53] Verschoyle R. D., Steward W. P., Gescher A. J. (2007). Putative cancer chemopreventive agents of dietary origin-how safe are they?. *Nutrition and Cancer*.

[54] National Toxicology Program (1993). NTP toxicology and carcinogenesis studies of turmeric oleoresin (CAS No. 8024-37-1) (major component 79%-85% curcumin, CAS No. 458-37-7) in F344/N rats and B6C3F1 mice (feed studies). *National Toxicology Program Technical Report Series*.

[55] Dance-Barnes S. T., Kock N. D., Moore J. E. (2009). Lung tumor promotion by curcumin. *Carcinogenesis*.

[56] Jiao Y., Wilkinson J., Di X. (2009). Curcumin, a cancer chemopreventive and chemotherapeutic agent, is a biologically active iron chelator. *Blood*.

[57] Appiah-Opong R., Commandeur J. N. M., van Vugt-Lussenburg B., Vermeulen N. P. E. (2007). Inhibition of human recombinant cytochrome P450s by curcumin and curcumin decomposition products. *Toxicology*.

[58] Thapliyal R., Maru G. B. (2001). Inhibition of cytochrome P450 isozymes by curcumins *in vitro* and *in vivo*. *Food and Chemical Toxicology*.

[59] Kurien B. T., Singh A., Matsumoto H., Scofield R. H. (2007). Improving the solubility and pharmacological efficacy of curcumin by heat treatment. *Assay and Drug Development Technologies*.

[60] Kurien B. T., Scofield R. H. (2009). Heat-solubilized curcumin should be considered in clinical trials for increasing bioavailability. *Clinical Cancer Research*.

[61] Anand P., Kunnumakkara A. B., Newman R. A., Aggarwal B. B. (2007). Bioavailability of curcumin: problems and promises. *Molecular Pharmaceutics*.

[62] Prasad S., Tyagi A. K., Aggarwal B. B. (2014). Recent developments in delivery, bioavailability, absorption and metabolism of curcumin: the golden pigment from golden spice. *Cancer Research and Treatment*.

[63] Matsui T., Yamane J., Mogi N. (2012). Structural reorganization of the bacterial cell-division protein FtsZ from *Staphylococcus aureus*. *Acta Crystallographica Section D: Biological Crystallography*.

[64] Singh P., Panda D. (2010). FtsZ inhibition: a promising approach for anti-staphylococcal therapy. *Drug News and Perspectives*.

[65] Gul P., Bakht J. (2015). Antimicrobial activity of turmeric extract and its potential use in food industry. *Journal of Food Science and Technology*.

[66] Gupta A., Mahajan S., Sharma R. (2015). Evaluation of antimicrobial activity of *Curcuma longa* rhizome extract against *Staphylococcus aureus*. *Biotechnology Reports*.

[67] Foweraker J. E., Laughton C. R., Brown D. F., Bilton D. (2009). Comparison of methods to test antibiotic combinations against heterogeneous populations of multiresistant *Pseudomonas aeruginosa* from patients with acute infective exacerbations in cystic fibrosis. *Antimicrobial Agents and Chemotherapy*.

[68] Sen G. S., Mohanty S., Hossain D. M. S. (2011). Curcumin enhances the efficacy of chemotherapy by tailoring p65NF*κ*B-p300 cross-talk in favor of p53–p300 in breast cancer. *The Journal of Biological Chemistry*.

[69] Ruiz de Porras V., Bystrup S., Martínez-Cardús A. (2016). Curcumin mediates oxaliplatin-acquired resistance reversion in colorectal cancer cell lines through modulation of CXC-Chemokine/NF-*κ*B signalling pathway. *Scientific Reports*.

[70] Sivanantham B., Sethuraman S., Krishnan U. M. (2016). Combinatorial effects of curcumin with an anti-neoplastic agent on head and neck squamous cell carcinoma through the regulation of EGFR-ERK1/2 and apoptotic signaling pathways. *ACS Combinatorial Science*.

[71] Wahl H., Tan L., Griffith K., Choi M., Liu J. R. (2007). Curcumin enhances Apo2L/TRAIL-induced apoptosis in chemoresistant ovarian cancer cells. *Gynecologic Oncology*.

[72] Saha S., Adhikary A., Bhattacharyya P., Das T., Sa G. (2012). Death by design: where curcumin sensitizes drug-resistant tumours. *Anticancer Research*.

[73] Nabekura T. (2010). Overcoming multidrug resistance in human cancer cells by natural compounds. *Toxins*.

[74] Köck R., Becker K., Cookson B. (2010). Methicillin-resistant *Staphylococcus aureus* (MRSA): burden of disease and control challenges in Europe. *Euro Surveillance*.

[75] Peacock S. J., Paterson G. K. (2015). Mechanisms of methicillin resistance in *Staphylococcus aureus*. *Annual Review of Biochemistry*.

[76] Balan P., Mal G., Das S., Singh H. (2016). Synergistic and additive antimicrobial activities of curcumin, Manuka honey and whey proteins. *Journal of Food Biochemistry*.

[77] Sharma G., Raturi K., Dang S., Gupta S., Gabrani R. (2014). Combinatorial antimicrobial effect of curcumin with selected phytochemicals on *Staphylococcus epidermidis*. *Journal of Asian Natural Products Research*.

[78] Chaieb K., Kouidhi B., Jrah H., Mahdouani K., Bakhrouf A. (2011). Antibacterial activity of Thymoquinone, an active principle of *Nigella sativa* and its potency to prevent bacterial biofilm formation. *BMC Complementary and Alternative Medicine*.

[79] Joung D.-K., Joung H., Yang D.-W. (2012). Synergistic effect of rhein in combination with ampicillin or oxacillin against methicillin-resistant Staphylococcus aureus. *Experimental and Therapeutic Medicine*.

[80] Lee Y.-S., Kang O.-H., Choi J.-G. (2010). Synergistic effect of emodin in combination with ampicillin or oxacillin against methicillin-resistant *Staphylococcus aureus*. *Pharmaceutical Biology*.

[81] Kang H.-K., Kim H.-Y., Cha J.-D. (2011). Synergistic effects between silibinin and antibiotics on methicillin-resistant *Staphylococcus aureus* isolated from clinical specimens. *Biotechnology Journal*.

[82] Joshi P., Singh S., Wani A. (2014). Osthol and curcumin as inhibitors of human Pgp and multidrug efflux pumps of *Staphylococcus aureus*: reversing the resistance against frontline antibacterial drugs. *MedChemComm*.

[83] Akiyama H., Fujii K., Yamasaki O., Oono T., Iwatsuki K. (2001). Antibacterial action of several tannins against *Staphylococcus aureus*. *Journal of Antimicrobial Chemotherapy*.

[84] Zhao W.-H., Hu Z.-Q., Okubo S., Hara Y., Shimamura T. (2001). Mechanism of synergy between epigallocatechin gallate and *β*-lactams against methicillin-resistant *Staphylococcus aureus*. *Antimicrobial Agents and Chemotherapy*.

[85] Gupta S. C., Patchva S., Aggarwal B. B. (2013). Therapeutic roles of curcumin: lessons learned from clinical trials. *AAPS Journal*.

[86] Burgos-Morón E., Calderón-Montaño J. M., Salvador J., Robles A., López-Lázaro M. (2010). The dark side of curcumin. *International Journal of Cancer*.

[87] McNally S. J., Harrison E. M., Ross J. A., Garden O. J., Wigmore S. J. (2007). Curcumin induces heme oxygenase 1 through generation of reactive oxygen species, p38 activation and phosphatase inhibition. *International Journal of Molecular Medicine*.

[88] Kang J., Chen J., Shi Y., Jia J., Zhang Y. (2005). Curcumin-induced histone hypoacetylation: the role of reactive oxygen species. *Biochemical Pharmacology*.

[89] Atsumi T., Fujisawa S., Tonosaki K. (2005). Relationship between intracellular ROS production and membrane mobility in curcumin- and tetrahydrocurcumin-treated human gingival fibroblasts and human submandibular gland carcinoma cells. *Oral Diseases*.

[90] Sharma R. A., Euden S. A., Platton S. L. (2004). Phase I clinical trial of oral curcumin: biomarkers of systemic activity and compliance. *Clinical Cancer Research*.

[91] Balaji S., Chempakam B. (2010). Toxicity prediction of compounds from turmeric (*Curcuma longa* L). *Food and Chemical Toxicology*.

[92] Manju S., Sreenivasan K. (2011). Conjugation of curcumin onto hyaluronic acid enhances its aqueous solubility and stability. *Journal of Colloid and Interface Science*.

[93] Dey S., Sreenivasan K. (2014). Conjugation of curcumin onto alginate enhances aqueous solubility and stability of curcumin. *Carbohydrate Polymers*.

[94] Esmaili M., Ghaffari S. M., Moosavi-Movahedi Z. (2011). Beta casein-micelle as a nano vehicle for solubility enhancement of curcumin; food industry application. *LWT—Food Science and Technology*.

[95] O'Toole M. G., Henderson R. M., Soucy P. A. (2012). Curcumin encapsulation in submicrometer spray-dried chitosan/Tween 20 particles. *Biomacromolecules*.

[96] Ucisik M. H., Küpcü S., Schuster B., Sleytr U. B. (2013). Characterization of CurcuEmulsomes: nanoformulation for enhanced solubility and delivery of curcumin. *Journal of Nanobiotechnology*.

[97] Pan K., Zhong Q., Baek S. J. (2013). Enhanced dispersibility and bioactivity of curcumin by encapsulation in casein nanocapsules. *Journal of Agricultural and Food Chemistry*.

[98] Thomas C., Pillai L. S., Krishnan L. (2014). Evaluation of albuminated curcumin as soluble drug form to control growth of cancer cells *in vitro*. *Journal of Cancer Therapy*.

[99] Kim T. H., Jiang H. H., Youn Y. S. (2011). Preparation and characterization of water-soluble albumin-bound curcumin nanoparticles with improved antitumor activity. *International Journal of Pharmaceutics*.

[100] Yallapu M. M., Jaggi M., Chauhan S. C. (2012). Curcumin nanoformulations: a future nanomedicine for cancer. *Drug Discovery Today*.

[101] Yallapu M. M., Jaggi M., Chauhan S. C. (2013). Curcumin nanomedicine: a road to cancer therapeutics. *Current Pharmaceutical Design*.

[102] Ghalandarlaki N., Alizadeh A. M., Ashkani-Esfahani S. (2014). Nanotechnology-applied curcumin for different diseases therapy. *BioMed Research International*.

[103] Quitschke W. W. (2008). Differential solubility of curcuminoids in serum and albumin solutions: implications for analytical and therapeutic applications. *BMC Biotechnology*.

